# Sex Differences in Salt Appetite: Perspectives from Animal Models and Human Studies

**DOI:** 10.3390/nu15010208

**Published:** 2023-01-01

**Authors:** Jessica Santollo, Derek Daniels, Micah Leshem, Jay Schulkin

**Affiliations:** 1 Department of Biology, University of Kentucky, Lexington, KY 40506, USA; 2 Department of Biology, University at Buffalo, Buffalo, NY 14260, USA; 3 School of Psychological Sciences, The University of Haifa, Haifa 3498838, Israel; 4 School of Medicine, University of Washington, Seattle, WA 98195, USA

**Keywords:** sex differences, sodium appetite, thirst, fluid homeostasis, motivated behavior

## Abstract

Salt ingestion by animals and humans has been noted from prehistory. The search for salt is largely driven by a physiological need for sodium. There is a large body of literature on sodium intake in laboratory rats, but the vast majority of this work has used male rats. The limited work conducted in both male and female rats, however, reveals sex differences in sodium intake. Importantly, while humans ingest salt every day, with every meal and with many foods, we do not know how many of these findings from rodent studies can be generalized to men and women. This review provides a synthesis of the literature that examines sex differences in sodium intake and highlights open questions. Sodium serves many important physiological functions and is inextricably linked to the maintenance of body fluid homeostasis. Indeed, from a motivated behavior perspective, the drive to consume sodium has largely been studied in conjunction with the study of thirst. This review will describe the neuroendocrine controls of fluid balance, mechanisms underlying sex differences, sex differences in sodium intake, changes in sodium intake during pregnancy, and the possible neuronal mechanisms underlying these differences in behavior. Having reviewed the mechanisms that can only be studied in animal experiments, we address sex differences in human dietary sodium intake in reproduction, and with age.

## 1. Introduction

Salt Appetite: The phenomenon of salt ingestion by animals and humans has been repeatedly noted in human history under a variety of conditions. Indeed, early recorded history from thousands of years ago contains reports of ingestion of “mineral” deposits or bone, presumably because of a hunger for salt [1,2]. For humans, salt (NaCl) is frequently ingested, every day, with every meal, and with many foods. Moreover, “salt cravings” have been reported by researchers and physicians under a wide range of clinical scenarios [3,4,5,6]. This penchant for salt likely arises from older times during which the primary means to preserve food was salt. However, the desire to consume salt is not limited to humans. Adequate sodium is essential for life in animals as well as humans, so it seems obvious how a liking for salt would proliferate in the species.

Elephants have been documented to search for deposits of salt. Many naturalistic studies have noted large risks taken by animals, mostly herbivores, to reach sources of sodium and other minerals to consume salt [1,2]. For instance moose, caribou, deer, reindeer, cattle and sheep are known to frequent and ingest sources of sodium at times when the sources of sodium are scarce in their natural habitat and the body’s chemical signaling systems (e.g., aldosterone, angiotensin) for sodium conservation are elevated [1,7,8].

This search for salt often puts animals in precarious positions, either because of the terrain in which the salt is found or because of increased risk of predation. The phenomenon is not just confined to herbivores. For instance, many bird species (e.g., finches, Red Crossbills, vultures) are known to frequent salt licks during periods of extracellular fluid needs [9]. Additionally, both mammals and many bird species frequent these sites during reproduction. Even chimpanzees [10] and gorillas [11] are known to frequent salt rich regions. This search for salt is largely driven by a physiological need for sodium (Na^+^). Thus, for scientific rigor, we refer to sodium appetite or sodium as proven in animal experiments, and ‘salt’ or ‘salt appetite’ for animals seeking sodium-bearing minerals, humans seeking NaCl exclusively, and as a generic term.

Sodium appetite is innate [1,12,13]; depleted rats (but also sheep) ingest sodium within milliseconds upon first exposure [12,14] and the appetite is expressed in suckling rats [15]. They also demonstrate an innate hedonic shift in the perception of sodium the very first time they are rendered sodium deplete [16]. Moreover, this recognition of the importance of sodium also occurs during times when sodium is at appropriate levels in the body. Indeed, sodium, even in the absence of a deficiency, has been shown to be a significant stimulus and able to facilitate diverse forms of learning (e.g., latent learning, sensory preconditioning [17,18]).

Importantly, gustatory mechanisms tied to the detection of sodium are found in invertebrates as well as vertebrates [19,20]. The circuit underlying salt taste in mammals has been well described [21,22] and appears to begin with the detection of sodium by diffusion of sodium ions into taste receptor cells through epithelial sodium channels (ENaC), although the necessity of traditionally described assemblies of ENaC has been called into question recently [23]. Signals from the gustatory afferents follow a classically ascribed pathway through the hindbrain that ascends to the gustatory cortex [24]. Components of these systems are highly conserved and comprise elements that are dedicated to the detection and identification of sodium and its sources [19,25]. Indeed, remembering sources where sodium can be found confers a large advantage for an animal in need of sodium [26]. This is especially important because sodium regulation is essential for both intracellular signaling systems and for extracellular fluid stability [1,27]. It is the latter that ties sodium intake broadly to fluid homeostasis. It is not surprising, therefore, that there are partially overlapping neural systems that respond to and control intake of both water and sodium. Thus, even though we humans might more commonly associate salt with our culinary experiences (and therefore consider it a part of food intake), from a physiological perspective, salt is far more relevant to fluid balance than it is to energy balance, placing it squarely in the purview of scientists who focus on drinking, more so than for those who focus on feeding.

Sodium appetite can be thought of in three contexts: the demands of extracellular fluid regulation, the demands of reproduction, both pregnancy and lactation, and the demands of stress related events. Despite the large body of literature on the sodium intake that develops in laboratory rats after depletion, the vast majority of this work has been limited to male rats. Accordingly, it remains unclear how much of these findings can be generalized to female rats, and perhaps even more important, how many of these findings can be generalized to men and women. Despite the limited use of female subjects, research that includes sex as a biological variable has identified sex differences in sodium intake. Hence, the main focus of this review provides a synthesis of the literature that examines sex differences in sodium intake, in relation to its demands of extracellular fluid regulation and the demands of reproduction, and highlights open questions remaining for the field to answer.

## 2. Neuroendocrine Controls of Fluid Balance

**Figure 1:**
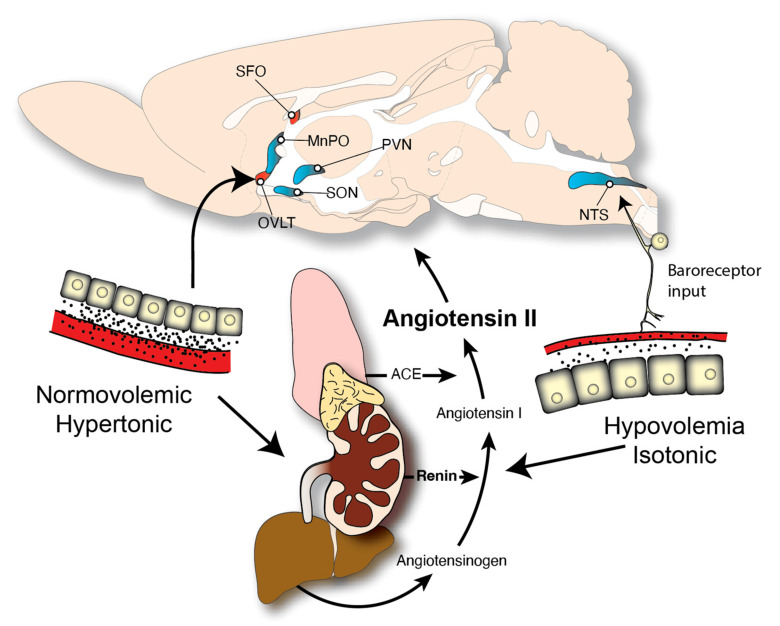
The renin-angiotensin system and its role in the double-depletion model of thirst and sodium intake. The renin-angiotensin system engaged by fluid loss. The active peptide of this system, angiotensin II, is generated by a biosynthetic pathway beginning with angiotensinogen, a relatively large liver protein that is abundant in circulation. Upon activation of the renin-angiotensin system, the kidney secretes renin, which cleaves angiotensinogen to form angiotensin I. Angiotensin I is further cleaved by angiotensin converting enzyme (ACE), which is expressed mainly in the lungs, to form angiotensin II, which acts on a variety of targets, including the brain. The stimuli that trigger thirst, and in some cases an accompanying sodium appetite, can be divided into intracellular and extracellular effects. In the case of intracellular dehydration, the extracellular volume remains constant in terms of volume (normovolemic), but with a greater concentration of solutes (hypertonic). In this case, the cells lose volume by osmosis, and this triggers activity of osmoreceptors in the brain, primarily in the OVLT, as well as activation of the renin-angiotensin system. In the case of extracellular dehydration, fluid and solutes are lost from the extracellular space. The concentration remains normal (isotonic), so there is no loss of water from the intracellular space, but the volume of the extracellular compartment is reduced (hypovolemia). The loss of volume and blood pressure engages hindbrain-projecting baroreceptors that synapse on cells in the nucleus of the solitary tract (NTS), and engages the renin-angiotensin system. Brain structures discussed in this article are shown. Structures in red indicate circumventricular organs that lack a blood–brain barrier and serve as a primary site of action for circulating AngII. Abbreviations: median preoptic nucleus, MnPO; organum vasculosum of the lamina terminalis, nucleus of the solitary tract, NTS; OVLT; paraventricular nucleus of the hypothalamus, PVN; subfornical organ, SFO; supraoptic nucleus, SON. Elements of the figure are recreated based on [28] and modified from [30].

Although sodium serves many important and specific physiological functions, it is inextricably linked to the maintenance of body fluid homeostasis. Indeed, from a motivated behavior perspective, the drive to consume sodium has largely been studied by researchers in conjunction with the study of thirst. Additionally, far more attention has been given to circuits underlying the need for sodium (and water) when there is a deficit in either or both, but animals often consume water and sodium in the absence of need. This need-free intake may occur because of a learned anticipation of need in the absence of intake, but more work is warranted to understand how this occurs. Nevertheless, our understanding of the mechanisms that drive thirst and sodium appetite during times of need has been heavily influenced by the double-depletion hypothesis proposed separately, but in the same volume, by Epstein and Fitzsimons [28,29]. This framework, illustrated in Figure 1, is built upon a long-standing and common understanding that mammals are mostly water, and that most of this water is found inside the cells of animals. The extracellular component is further compartmentalized into water found in the blood and the water outside of circulation that bathes the tissues of the body. The crux of the double-depletion hypothesis, as proposed, was that depletion of either the intracellular or extracellular compartments resulted in thirst, but the mechanisms required for detecting these separable depletions, and the behaviors needed to address them, were distinct. Specifically, depletion of the intracellular space is detected by osmoreceptors and leads to water intake, whereas extracellular depletion is detected by baroreceptors and sensory elements of the kidney, resulting in intake of both water and sodium.

Angiotensin II (AngII) is a key hormone in the control of thirst and sodium appetite. Application of exogenous AngII to the forebrain cerebral ventricles of rats is so reliable in the water intake it causes that it is often used as a means to verify accurate cannula placement in the ventricle. AngII is also capable of inducing sodium appetite. In studies of laboratory rats, sodium intake induced by AngII occurs in two phases. The first of these phases can be induced by AngII alone and involves a rapid increase in sodium intake. Thus, intake will occur rapidly, but generally only when more dilute, yet somewhat hypertonic (e.g., 1.5%), saline solutions are offered. The second phase, however, results in what has been referred to as a true appetite. This distinction between intake with or without appetite has been used to differentiate between the drive to consume lower concentration saline solutions and the change in acceptance and consumption of strongly hypertonic saline solutions (e.g., 3%). Although administration of AngII can induce this second phase of intake, it requires relatively high doses that are clearly outside of the physiological range. When co-administered with a substance that engages mineralocorticoid receptors, however, much lower doses of AngII are needed, and the combination of AngII and mineralocorticoid results in intake that is greater than the additive intake occurring from each treatment individually [31,32].

Mineralocorticoid hormones (e.g., aldosterone, deoxycorticostone) are the other principal hormones in sodium restoration, both for conservation and redistribution of sodium in extracellular fluid regulation and in acting in the brain in generating the hunger for sodium [33,34,35,36] Interestingly, the steroid may be critical for the hedonic shift. Pharmacological blockade of MR receptors blocks or reduces the hedonic shift in the oral facial profile associated with sodium hunger in sodium depleted rats [37,38].

**Figure 2:**
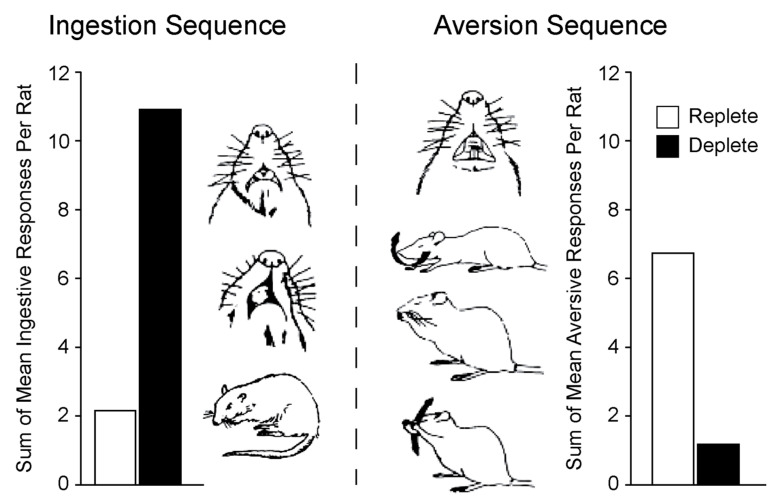
Sodium depletion changes taste responses to hypertonic saline. Data previously published in table form [16] were used to calculate mean ingestive and aversive responses after oral infusion of 3% NaCl. Sodium depletion caused a clear shift in the relative number of ingestion and aversion sequences of behavior. Drawings are from [45]. The figure was reproduced, with permission, from [46].

A full understanding of the change in the system that causes this altered state remains incomplete. In his early studies on the sodium appetite that occurs after adrenalectomy, Richter posited that this change in behavior likely involved an increased sensitivity to sodium that would help animals find sources of sodium that were necessary for survival, especially in the deplete state [39,40]. Although this seems intuitive, experiments using recordings of taste afferents found a *decrease* in the sensitivity of sodium-sensitive fibers associated with sodium deprivation [41]. This change in sensitivity is consistent with changes in ingestive and aversive responses to intraoral infusion of 3% saline and taste-reactivity testing (Figure 2), in which aversive responses decrease and ingestive responses increase in sodium deplete rats. These changes appear to be mediated by endogenous endorphins [38,42].

Although these two approaches suggest that the perception of sodium by a sodium deplete rat is blunted, such that high concentration saline that is normally disliked becomes liked, brief access tests using a Davis Rig [43] failed to support the hypothesis that there is a shift in the preference curve of sodium concentrations. Specifically, using this approach, sodium deplete rats were found to lick all solutions more, instead of showing a shift toward more licking of high concentration solutions [44]. Accordingly, it remains unclear how the shift in taste reactivity and nerve recordings can be reconciled with the lack of a shift in brief-access testing, but the avid consumption of sodium by the sodium deplete rat is undisputed.

In spite of the large body of literature on the sodium intake that develops in laboratory rats after depletion, the vast majority of this work has been conducted using male rats. Accordingly, it remains unclear how much of these findings can be generalized to female rats, and perhaps even more important, how many of these findings can be generalized to men and women. The remainder of this review is intended to provide a synthesis of the literature that examines sex differences in sodium intake.

## 3. Mechanisms Underlying Sex Differences

**Figure 3:**
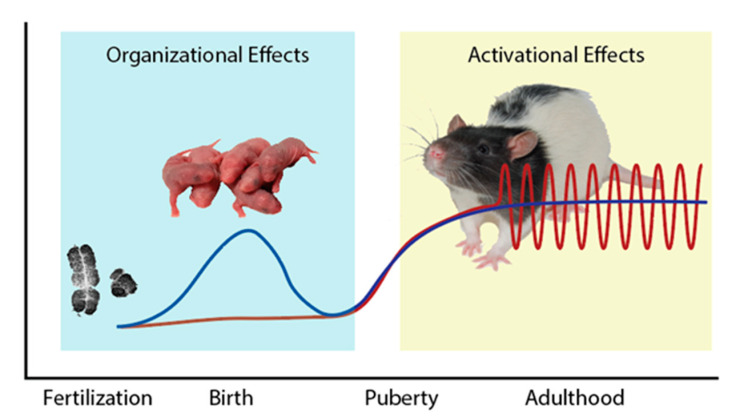
Endocrine changes associated with life stages in the rat. A surge in testosterone (blue line) occurs during the perinatal period in male rats, causing many organizing effects. Levels of estradiol (red line) are relatively low during this time, in both male and female rats. At puberty, however, levels of these hormones begin to rise with levels of testosterone increasing in male rats and levels of estradiol rise in female rats, leading to the activational effects of these hormones in their respective sexes. Levels of testosterone remain relatively stable, whereas levels of estradiol become pulsatile. These organizational-activational effects underlie many sex differences in behavior. Sex chromosome complement, determined at fertilization, also influences several sex differences in the adult rat.

To understand the mechanisms underlying sex difference in sodium intake, one first needs to consider the variety of ways gonadal hormones and sex mediate differences between males and females. The most seemingly straightforward mechanism by which gonadal hormones can underlie sex differences is through “activational” effects in adulthood. The exposure to differential levels of estrogens, progestins, and/or androgens between males and females in adulthood can result in sex differences in physiology and behavior. These differences are temporary and present only during hormone exposure. Either removal of the circulating hormone or exogenous administration of the hormone to the opposite sex will mitigate the sex difference. Sex differences in locomotor activity is a classic example of an activational effect of estradiol. In most mammals, females are more active than males and an easy way to observe this behavior in the laboratory is through quantification of revolutions run on a wheel [47,48]. Ovariectomy eliminates the observed sex difference in wheel running [49], and exogenous estradiol treatment increases running wheel activity, to a similar degree, in ovariectomized (OVX) females, castrated males, and masculinized (see below) females [48,49]. This suggests that the sex difference in activity is caused by different levels of circulating estrogens in males and females. Next, sex differences can result from different developmental exposure to gonadal hormones, during a critical period, that results in a masculinized or feminized brain. These are referred to as the organizational effects of gonadal hormones. This critical period was originally defined as the time in utero and/or just after birth when males are exposed to a burst of testosterone secretion, while females are not exposed to gonadal hormones. More recent studies, however, identify puberty as a second critical period for the organizing effects of gonadal hormones [50]. These organizing effects of gonadal hormones result in permanent changes in brain structure and function. Treatment of females with testosterone at birth masculinizes (or defeminizes) brain architecture, while castrating males at birth, or treatment with an androgen receptor antagonist does the opposite, producing a feminized/demasculinized brain. Sex differences in juvenile play behavior [51], which occur prior to high levels of circulating gonadal hormones that begin a puberty, is one example of a sex difference that is organized by gonadal steroids. Next, the foundational work on the mechanisms underlying sex differences comes from Young and colleagues on reproductive behavior in the 1950s. Their classic work on male and female typical reproductive postures illustrates that sex differences can require both activational and organizational effects of gonadal hormones. For these behaviors, sex hormones in adulthood act on masculinized or feminized brain structures (that were organized during development by gonadal hormones or lack thereof) to produce the sex specific behavior, in this case mounting in males and lordosis in females [52]. Finally, sex differences in physiology and behavior can be independent of gonadal hormones. Differences in sex chromosome complement are one mechanism. The development of the four-core genotype mouse model in the early 2000s, which allows for the decoupling of gonadal sex and sex chromosomes, has advanced our understanding of the contribution of sex chromosomes in mediating sex differences over the past 20 years [53]. Figure 3 summaries the different biological mechanisms underlying sex differences. Additionally, the effect of the environment, and in particular, effects of the environment during critical periods of development can contribute to sex differences. In humans, social and cultural factors can also underlie sex differences in behavior.

## 4. Sex Differences in Unstimulated/Need-Free Sodium Intake

Daily unstimulated (also referred to as ab libitum or need-free) saline intake is greater in adult female rats, compared to adult male rats, and to our knowledge there are no discrepancies in the literature on the direction of this sex difference. Taste reactivity tests (observing face, limb and body movement responses) demonstrate that females have more ingestive responses to sodium than males, and make more licks in the Davis Rig (which eliminates postingestive feedback signals) for sodium than males [54,55,56,57,58,59,60]. Together this suggests that differences in gustatory sensitivity contribute to the sex difference in sodium intake. There are discrepancies, however, regarding the mechanism(s) by which gonadal hormones mediate this sex difference. The sex difference in intake has been reported in rats as young as 30 days of age, which is prior to high levels of circulating gonadal hormones associated with puberty [58]. In addition, the sex difference in intake is present in adult males and females gonadectomized at 10 days of age [55]. In two bottle preference tests, both control and estradiol-treated OVX females consume more saline and have a higher saline preference than males [58,59,60]. These studies suggest that adult gonadal hormones are not necessary for expression of the sex difference and therefore the preferences are mediated by either organizational effects of gonadal hormones or non-hormonal factors such as sex chromosome complement. Three studies, to our knowledge, have directly addressed the role of organizational effects of gonadal hormones in mediating the sex difference in sodium intake. Females masculinized/defeminized at birth, via testosterone treatment, have male like levels of ab libitum saline intake and males castrated a birth, resulting in a feminized/demasculinized brain, have enhanced saline intake in adulthood [54,61]. This work was confirmed and extended by Chow and colleagues who showed that the masculinization/defeminization of saline intake occurs through activation of estrogen receptors. Females treated at birth with testosterone, but not the nonaromatizable dihydrotestosterone, had male like levels of saline intake in adulthood [57].

This seemingly straightforward explanation, that organizational effects of gonadal hormones underly the sex difference in unstimulated saline intake, is challenged by conflicting reports on the age at which the sex difference is first observed. An earlier report by Krecek, did not observe a sex difference in saline intake until 60 days of age (adulthood; [55]). Furthermore, Chow et al. observed a small difference in saline intake between males and females at 45 days of age, which became significantly pronounced by 55 days of age [57]. These reports, therefore, suggest that activational effects of gonadal hormones at least play a role in the sex difference. Considering, however, the complicated relationship between fluid intake, body weight, and sex [62,63], it is also possible that sex differences in saline intake are present prior to puberty but difficult to observe due to rapid changes in body weight that occur at different rates between males and females. In support of this idea, castration at birth increased saline intake in males starting at 85 days of age, while neonatal testosterone treatment reduced saline intake in females as early as 30 days of age (intake measurements in this study were not collected earlier, see [54,61]). Again, differences in body weight and the differential relationship between body weight and fluid intake in males and females may mask sex difference in saline intake during times of rapid growth [63,64]. Another possibility is that organizational effects of gonadal hormones are needed to form the appropriate masculinized or feminized brain circuitry, which are then acted upon in adulthood by non-gonadal hormones. In support of this idea, pinealectomy enhances the natriorexigenic effect of neonatal castration in adult males [61,65]. To our knowledge, the role of pineal hormones on saline intake in females has never been tested.

Gonadal hormones also have activational effects on unstimulated saline intake in adult males and females, which may be independent of any role related to sex differences in intake. In females, saline intake fluctuates across the estrous cycle, with the lowest intake on the day of estrus [66]. This suggests that ovarian hormone exposure in adulthood acutely reduces saline intake. Indeed, low doses of estradiol administered to OVX adult rats reduces saline intake [67]. However, high doses of estradiol increase saline intake in both female and male rats [1,67,68]. While initially surprising, recent reports highlight bidirectional effects of estradiol on water and food intake and this may be another example of paradigm dependent, directional effects of estradiol on ingestion [69,70]. In addition to behavioral differences, corda timpani responses to saline are greater in males and OVX-oil (control)-treated females, compared to OVX-estradiol-treated females [59]. This suggests that differences in gustatory sensitivity may contribute to hormonally mediated, acute changes in saline intake. Finally, Chow and colleagues demonstrated that although gonadectomy in adulthood had no effect on saline intake in either sex, exogenous testosterone treatment in castrated males and OVX females reduced saline intake [57]. It is difficult to reconcile the null effect of castration with the anti-natriorexigenic effect of exogenous testosterone treatment and to our knowledge this is the only study to examine the effect of testosterone in adulthood on saline intake. One possibility, however, is that the exogenous testosterone is aromatized to estradiol which then reduces intake. This hypothesis needs to be directly tested to understand the activational role of testosterone on unstimulated saline intake.

To summarize, ad libitum saline intake is greater in females than males, and early life exposure to gonadal hormones have organizational effects that, at least in part, mediate this sex difference. Gonadal hormones in adulthood, however, also influence saline intake but whether and how these effects contribute to the sex difference is unclear.

## 5. Sex Differences in Stimulated Sodium Intake

Although there are multiple reports demonstrating sex differences in stimulated sodium intake, there are inconsistencies in the direction of this difference. Central injections of renin and paradigms using furosemide treatment in combination with a sodium deficient diet for 1–2 days typically produces greater sodium intake in female, compared to male, rats [15,57,71,72,73], but see [74]. However, maintenance on a sodium deficient diet for eight days generates greater sodium appetite in males, compared to females [75,76]. The same result was reported in rats subjected to a sodium deficient diet for four days and then treated with the colloid polyethylene glycol [76]. These discrepancies could be related to the degree of sodium deficiency. Potentially, in paradigms that induce a more acute sodium loss (1–2 days), the sodium intake is greater in females, compared to males. In paradigms, however, with a more chronic sodium loss (>2 days), the sodium appetite is greater in males. To reconcile these differences, this hypothesis needs to be tested. To our knowledge, only one study has compared sodium appetite between male and female mice. An acute sodium loss, induced by furosemide treatment plus 21 h of sodium deficient diet, resulted in greater sodium appetite in males, compared to females [77], the opposite direction of that in the rat [57,71]. Species-specific differences, therefore, also need to be considered when attempting to reconcile the discrepancies in the literature on sex differences in sodium appetite.

Despite multiple behavioral reports demonstrating sex differences in sodium appetite, there is a dearth of studies aimed at understanding the underlying hormonal mechanism driving sex differences in sodium intake. The sex difference in renin-stimulated saline intake (F > M) was observed in rats 14–17 days old [15], suggesting that circulating gonadal hormones are not involved in the sex difference and either organizational effects of gonadal hormones or sex chromosome complement are involved. However, the sex difference in sodium intake induced by eight days of sodium deficient diet (M > F) was reported in 40 day-old, but not 30 day-old rats [76]. This suggests that circulating gonadal hormones are necessary for the sex difference, but whether a masculinized or feminized brain is also necessary for the circulating gonadal hormones to act on has not been tested in rats. To our knowledge only one study has directly tested the organizational role of gonadal hormones and sex chromosomes and it was the sole report in mice, where the direction of the sex difference is opposite that of the rat (M > F mice). Using the four-core genotype mouse model, organizational differences from developmental gonadal hormone exposure, and not sex chromosome complement, mediated the sex difference in sodium appetite [77]. Whether this sex difference is mediated by differential sensitivity to gustatory stimuli, similar to mechanisms observed in rats [54,55,56,57,58,59,60], has, to our knowledge, not been explored. Exploring sex differences in taste responses with mice lacking ENaC in taste buds will help address this question [78]. More research is needed in the rat to understand the role of activational and organizational effects of gonadal hormones and sex chromosome complement, and how these mechanisms may differ by the paradigm used to induce sodium appetite.

There are multiple reports of activational effects of gonadal hormones influencing stimulated sodium intake in males and females. Unlike the discrepancies related to the direction of the sex difference in stimulated saline intake, all available literature examining estradiol report an anti-natriorexigenic effect. AngII-stimulated saline intake varies across the estrous cycle with lowest intakes on the day of estrus, suggesting an inhibitory role of ovarian hormones [79,80]. Indeed, estradiol or estrogen receptor agonists reduce AngII-stimulated saline intake in OVX rats [81,82,83], an effect not modulated by co-treatment with progesterone [84]. Furosemide treatment plus 24 h of a low sodium diet produces greater sodium intake in diestrus, compared to estrus, females and in OVX-oil-treated (control treatment), compared to OVX-estradiol-treated, females [85]. The anti-natrorexigenic effect of estradiol is also observed in paradigms that induce a more chronic sodium loss. For example, OVX rats consume more sodium than intact females after 8 days of sodium deficiency. Estradiol-treatment reduces this intake in both OVX females and in castrated males [75,76]. Finally, sodium appetite is greater in male and OVX females, compared to intact female rats, after 7 days of deoxycorticosterone treatment [76]. Again, estradiol-mediated changes in gustatory processes likely contribute to the reduction in intake. In OVX rats, estradiol-treatment reduced hedonic responses and increased aversive responses to intra-oral infusion of sodium in rats subjected to a water deprivation, partial rehydration paradigm [86]. Little research has focused on the activational effects of testosterone on sodium appetite. Chow and colleagues reported that testosterone treatment decreased sodium intake in OVX females, but not castrated males, after sodium depletion induced by furosemide plus 24 h of a sodium deficient diet. Again, it is possible that the testosterone was aromatized to estradiol, resulting in the reduction in sodium intake in females and future studies will need to test this hypothesis. Again, how the activational effects of estradiol and testosterone contribute to the sex difference in stimulated sodium appetite is unclear, and future work is needed to understand these connections.

## 6. Changes in Sodium Intake during Pregnancy

Greater sodium intake in females, compared to males, may be an adaptive function, related to the need for sodium during gestation and lactation. Adequate sodium consumption during pregnancy is critical for fetal and offspring survival and health. Maintaining pregnant rats on a low sodium diet results in smaller liters, fewer live births, more stillbirths and fewer offspring surviving to weaning and low sodium in utero is associated with altered kidney function, lower brain protein levels, and increased blood pressure in the offspring [87,88]. It is, therefore, also not surprising that increased maternal sodium intake during late pregnancy and throughout lactation has been observed across many species including rats, mice, and rabbits [89,90,91,92,93], although this enhancement is not always observed if animals are not hypovolemic or sodium deficient [94]. This intake is unrelated to changes in sodium excretion, and hence not a behavioral compensation to offset sodium loss [89,90,95]. Adrenalectomy has no effect on sodium intake in pregnant rats, again suggesting that increased intake is unrelated to sodium loss [90]. Furthermore, the increased sodium content is not associated with tissue of either the dam or offspring but is sequestered in the uterus, at least in the rat [89,90]. While increased lick rates in the Davis Rig (which eliminates postingestive feedback) for sodium during late pregnancy and lactation suggest that gustatory changes underlie the enhanced sodium intake [96], others have reported no changes in either ingestive or aversive responses to sodium in pregnant dams during taste reactivity tests [93]. The altered hormonal milieu of pregnancy likely plays a key role in the increased sodium intake. Pseudo-pregnant rabbits and virgin rabbits treated daily with estradiol increase sodium intake, and while progesterone alone has no effect on intake, it enhances the natriorexigenic effect of estradiol [95]. Non-pregnant rabbits treated with doses of prolactin and oxytocin to mimic lactation increased sodium intake with no preceding change in urinary sodium excretion [97]. This again suggests that the increase in sodium during lactation is primarily mediated by hormones and not caused by a sodium deficiency due to lactation. Finally, pregnancy and/or its combination with lactation produces long lasting increases in sodium intake in the dam that persist beyond weaning, similar to that observed after sodium deprivation [98]. As discussed previously, there are mixed reports on the inhibitory vs. stimulatory effects of estradiol on ab libitum saline intake in nonpregnant females [67], which may be related to the dose of the hormone. This maybe an opportunistic effect of the estradiol, inhibiting intake during times of low sodium need, but enhancing intake during times of critical sodium need. More research is needed to understand how different levels of estrogens bidirectionally influence sodium intake and to understand the underlying mechanisms driving sodium intake that are altered by estrogens during pregnancy and lactation.

## 7. Neural Controls of Sodium Intake: Implications for Sex Differences

For a thorough understanding of how gonadal hormones and sex augment the neuronal controls of sodium intake and produce sex differences and increase intake during pregnancy/lactation, a complete knowledge of the neural circuitry that drives saline intake and sodium appetite is required. While our understanding of this circuit is still incomplete, three critical forebrain regions, which are also sexually dimorphic, have been identified. These brain regions are the medial preoptic region, the central/medial region of the amygdala, and the medial region of the bed nucleus of the stria terminalis. Angiotensin II action in the lamina terminalis [99,100], with projections to the bed nucleus of the stria terminalis [101], stimulates acute saline intake. In addition, aldosterone sensitive cells in the parabrachial nucleus (PB) and pre-locus coeruleus (pre-LC) send projections to several forebrain sites, including bed nucleus of the stria terminalis, the ventral tegmental region, paraventricular nucleus and central/ medial amygdala. These PB and pre-LC to forebrain projections are likely critical for both activating and satiating sodium appetite [102,103,104,105,106,107,108].

It is unclear how gonadal hormones and sex augment these neuronal pathways to influence saline intake/sodium appetite. The presence of estrogen receptors throughout these brain regions, however, provides a direct mechanism by which estradiol in adulthood, or aromatized testosterone in utero, can induce acute or permanent changes within these brain circuits [109]. Activation of estrogen receptor beta and G protein estrogen receptor 1 reduce AngII-stimulated saline intake in OVX rats, suggesting a key role of these receptors [81,82]. Furthermore, estradiol augments neuronal activity within the SFO and OVLT, but not the MnPO, during sodium depletion in OVX rats [85]. This provides a logical starting point for examining how estradiol augments the neuronal circuits controlling sodium intake. More work is required to understand the neuronal controls of sodium intake and sodium appetite. That work will be critical to guide research on elucidating how gonadal hormones control these pathways.

## 8. Human Salt Intake

The sodium ion is essential in humans as it is in other animals. The hormonal, physiological and brain systems regulating bodily sodium in humans and other animals are similar and both have dedicated taste organs for the sodium ion, and remarkably, in both, early sodium deficit programs increased avidity for salt throughout life [110,111]. Notably, the behavioral arm of these systems differs dramatically: humans do not have a salt appetite: where rats take sodium with a variety of anions [2,14,112], humans exclusively take NaCl (table salt); where experimental animals accept salt dissolved in water, these solutions may make humans retch [113]; where humans take it exclusively in food animals do not [114,115]; where animals lick its crystals [116,117], humans do not. Most tellingly, where hyponatremic animals seek and recognize the cure (sodium), hyponatremic humans may die with sodium at hand [118,119]. Hence, we suggest that humans may have no specific ‘sodium appetite’ as do insects, birds, rodents and ungulates, but the human insatiable predilection for salt in its multitude forms of ingestion in food may be termed a ‘salt appetite’. Yet, like animals, in humans there are notable sex differences in salt appetite, except that unlike female animals, it seems that women have the lesser salt appetite.

## 9. Sex Differences Related to Reproduction in Humans

**Figure 4:**
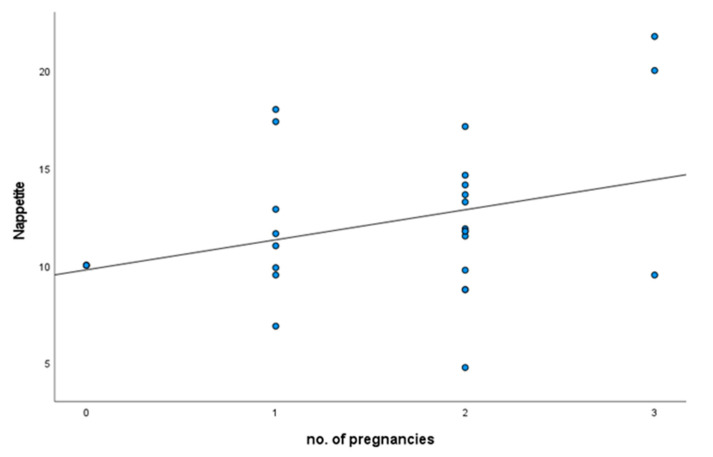
Salt appetite by number of pregnancies.

Adequate neonatal sodium is crucial for growth and cognitive development [120]. Postnatal sodium deficit has been linked to increased lifelong sodium intake, and maternal sodium preference to offspring life-long blood pressure [121,122]. In pregnant women it is not clear whether sodium intake is increased, despite a number of studies [96,123,124,125]. When comparing nursing mothers, mothers not-nursing, at up to 6 m post-partum, and nulliparous women, there were no differences in sodium preference or intake, salt appetite, sweet preference, other measures of salt and sweet preference, or intake of dietary electrolytes and macronutrients and no interaction was found with number of births (Leshem, unpublished data, Figure 4 and [126]). This is not unexpected insofar as in direct comparison to the animal studies. The nutritional demand that the human offspring place on nursing mothers is obviously less than the demand on the rat dam from a litter that can be twice the dam’s body weight. Nevertheless, the human sample was small, and power analysis suggests a correlation of number of births with salt appetite that would be detected with a larger sample. Clearly such a finding would be of interest. Moreover, throughout pregnancy women increasingly manifest a lower intensity sensory response but a greater hedonic response to sodium chloride compared to nonpregnant women [127]. The US Dietary Reference values for sodium and chloride [128] are similar for pregnant and lactating women and non-pregnant women—and men at every adult age (https://www.dietaryguidelines.gov/sites/default/files/2021-03/Dietary_Guidelines_for_Americans-2020-2025.pdf (accessed on 28 December 2022)). On the other hand, mothers reporting low salt preference persisted in breastfeeding beyond day 7 postnatal in comparison to mothers with high salt preference, who also had the shortest exclusive breastfeeding duration up to postnatal day 25 [129]. Can one speculate that low sodium makes babies encourage more or longer breastfeeding?

Unlike rats, for humans the evidence for variation with the reproductive cycle is inconsistent and has yet to be related to physiological sodium need [123,124,125,130,131,132,133,134,135,136].

## 10. Sex Differences in Dietary Sodium Intake

Salt intake is lower in women, which is in line with their lower caloric intake and body weight, so that either or both lean tissue and whole body utilize the sodium. Similarly, sodium intake is lower in women by 9–11% worldwide [137], suggested as a function of their lower caloric intake [128,138,139,140].

Analyses of NHANES data show that US men ingest 45.4 mg/d/kg body weight Na^+^ and women 39.0 mg/d/kg, vastly less. Similarly, Israeli men and women intake 41.25 and 33.91 mg/kg/d, respectively, an even greater difference of 20% so that for a 75 kg individual (mean weight for the sample), this is about 1.4 g/d more salt for a man than a woman [141]. Because the data do not include salting at the table, more salting by men increases the difference (below). Intriguingly, could it derive from gender specific dietary choices and differences in natriuresis, microbiota, and nutrient metabolism [142,143,144,145]? Clearly, the sex difference in intake of sodium, its causes, mechanisms, and implications, require further investigation.

## 11. Sex Differences in Salting of Foods

**Figure 5:**
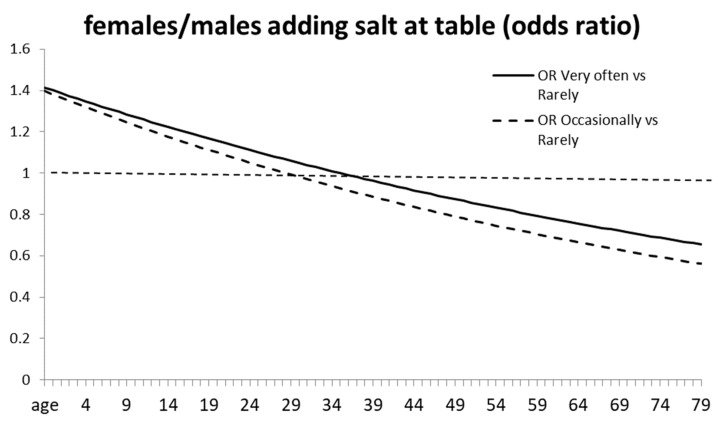
Odds ratios plotting responses to questions on frequency of adding salt by sex and age. Above the reference line at value 1 women salt more, below it, men salt more, so that the sex difference inverts with age. From [138].

In NHANES data, an age–gender interaction for adding table salt “very often” and ‘occasionally’ vs. ‘rarely’ indicates that below ~30 years of age women add salt more frequently than men, after which men add salt more (Figure 5). Estimates of added salt as 5% of total sodium intake in the US might marginalize the contribution of voluntary salt intake, but elsewhere, e.g., Italy, it may be 30%, and in China even more, so that the gender difference among younger adults might be significant in offsetting the higher dietary intake of men, and possibly exceeding it when corrected for bodyweight [146,147,148]. In a lab food choice study, British men (~26 years old) eat significantly more salty foods than women [149]. Although a report links dietary sodium and adding salt [150,151], others have not found such a consistent relationship [110,121,152,153,154].

**Figure 6:**
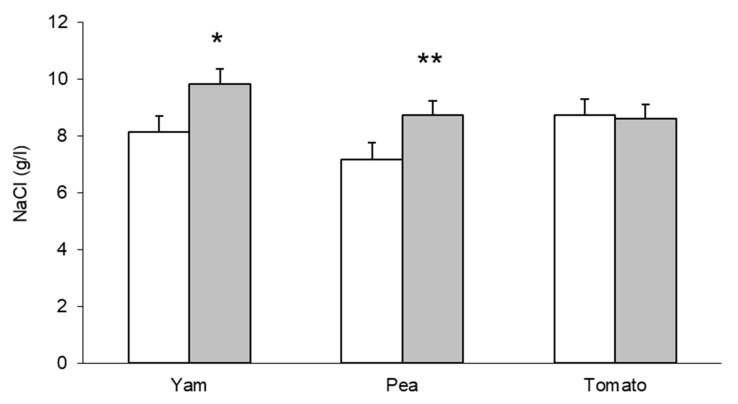
Sex difference in preferred salt concentration in soups. Women, white columns, men, grey columns, n = 41–110/column, * *p* > 0.02, ** *p* < 0.005, From [108].

Sex differences in salting may be confounded by taste—509 students were offered free cups of soup at a stall in the university corridor. The soup given to each student was salted at a concentration they chose from six samples of the soup differing in concentration of salt. Yam, pea, and tomato soups were tested on different days, women chose lower salt concentrations than men for yam and pea, but not tomato soup (Figure 6; [110]).

## 12. Sex Differences in Salt Intake in the Elderly

Like other topics discussed in this review, there has been a notable lack of attention to sex differences in salt intake in the elderly., which is of relevance given that the elderly are most at risk for disturbances in body fluid balance. In a Palestinian township, dietary Na^+^ intake recalled from 20 years previous, at middle age (45–58 y), was greater than the current intake at old age (65–85), related to greater caloric intake, and was greater in women. Interestingly, the older women BMI was greater but their sodium intakes did not differ, consistent with the lean body hypothesis of sodium intake, as well as suggesting a relationship of salt appetite to estrogens in these post-menstrual women [155]. In agreement with the literature on impaired thirst in elderly people [156], elderly participants did not report an increase in thirst during the 60–80 min test, unlike middle-aged participants. High sodium intake is associated with hypertension and as women age they become more likely to develop hypertension and associated CVD outcomes so that awareness of their increased sodium intake can focus prevention more effectively [157].

## 13. Conclusions and Discussion

Many species under a number of conditions seek out sources of sodium, often found in salt licks where there are diverse minerals [1,2]. Sodium is fundamental in the maintenance and restoration of extracellular fluid regulation. Indeed, sodium also serves as a fundamental gustatory signal [158]. Dedicated neural circuits underlie salt ingestion across life and during sodium need. Earlier anecdotal observations suggest elevated salt intake in humans under conditions of sodium need, and while there are clear differences in salt appetite that are impacted by biological sex and gonadal hormones in animal studies, it is much less clear in humans.

Salt is omnipresent in human consumption, unlike what other animals have to adapt to. Culture may mask regulatory capability, and in most cultures, there is a surfeit of salt in our diets. Indeed, while there are clear differences in salt appetite and salt intake with female rats demonstrably showing a greater avidity for salt solutions (except under extreme deprivation paradigms), the opposite occurs in humans with substantially greater salt intake in men. We do not know why humans like the taste of salt. One possibility is that salt has unknown benefits that condition a preference. If so, it is also likely that the benefits would differ by sex, for example women may add more salt when depressed as a result of conditioning by anti-depressant effects of sodium as are athletes by untasted salt after exertion [141,159].

Here, we presented the first and thorough review of the sex differences in salt appetite in both laboratory animals and humans. Sex differences in salt appetite are well documented in the laboratory rat with some understanding of its origins, determinants, endocrinological, physiological and neural mechanisms and its behavioral and reproductive underpinnings. There is also a human literature demonstrating sex differences in sodium intake, however the implications of these differences are weighted toward the health consequences of high salt intake and somewhat to reduction strategies. While the medical consequences of high salt intake for women are well known, there is modest attention for its implications in reproduction for women and its effects on women’s mood are scarcely studied. Finally, since salt is the only nutrient whose attraction, intake, disposition, and effects differ dramatically by sex, these differences demand increased focus on female animals to research the underlying mechanism, and increased consideration of the import of salt intake in women.

## Data Availability

Not applicable.
